# Continuous Infusion of Ketamine and Lidocaine Either with or without Maropitant as an Adjuvant Agent for Analgesia in Female Dogs Undergoing Mastectomy

**DOI:** 10.1155/2021/4747301

**Published:** 2021-01-26

**Authors:** Priscila C. L. R. Soares, Janaina M. X. Corrêa, Raquel V. Niella, Jéssica N. S. de Oliveira, Brenda A. Costa, Alex C. Silva Junior, Aline S. Sena, Taísa M. Pinto, Alexandre D. Munhoz, Luci Ana F. Martins, Elisângela B. Silva, Mário S. L. Lavor

**Affiliations:** ^1^Postgraduate Program in Science Animal, State University of Santa Cruz (UESC), Ilhéus, Bahia, Brazil; ^2^Collegiate of Veterinary Medicine, Department of Agricultural and Environmental Sciences, State University of Santa Cruz (UESC), Ilhéus, Bahia, Brazil; ^3^Department of Agricultural and Environmental Sciences, State University of Santa Cruz (UESC), Ilhéus, Bahia, Brazil

## Abstract

Maropitant, an antagonist of neurokinin-1 (NK-1) receptors, blocks the pharmacological action of substance P on the central and peripheral nervous systems. The objective of this study was to compare the antinociceptive and cardiorespiratory effects of the continuous intraoperative infusion of maropitant with ketamine and lidocaine in female dogs undergoing unilateral radical mastectomy. Twenty-four female dogs were used and were divided randomly into two groups (*n* = 12). The GLK group received ketamine bolus (1.0 mg/kg), lidocaine bolus (1.5 mg/kg), and continuous infusion of ketamine and lidocaine (10 mcg/kg/min and 50 mcg/kg/min), respectively; the GLKM group received the same anesthetic protocol combined with maropitant bolus (1.5 mg/kg/IV) and continuous infusion of maropitant (100 mcg/kg/h). Continuous infusion was initiated at the start of surgery and was maintained until 1 hour postoperatively. Pain was evaluated in the postoperative period using four scales and a digital analgesimeter. Data were analysed using analysis of variance, Student's *t*-test, Mann–Whitney test, and Friedman's test (*P* < 0.05). Kaplan–Meier curves were compared using the log-rank test. The results indicated lower pain scores, better survival curves with a lower number of patients requiring rescue analgesia, and lower peripheral sensitization, in the GLKM group than in the GLK group. It was concluded that the coadministration of maropitant with ketamine and lidocaine had an adjuvant effect with minimal cardiorespiratory effects and effective analgesia, improving pain management and patient comfort.

## 1. Introduction

Mammary neoplasms are common in small animals and usually affect uncastrated females or animals that have been castrated late [1, 2]. Mastectomy induces an exacerbated inflammatory response in the postoperative period [3]. The pain in this period may be attributed to some extent to the failure in analgesia techniques, compromising the success of patients' recovery and resulting in acute and chronic pain [1]. It is believed that the use of multimodal protocols improves patient comfort and decreases adverse drug effects [4].

Adjuvant analgesia techniques usually reduce the need for anesthetics, providing adequate pain management and minimal adverse effects. A technique used in mastectomy is intermittent bolus administration and continuous infusion of ketamine and lidocaine. This protocol provides a constant concentration of these drugs, faster recovery, and normalization of plasma levels [1, 5, 6].

The neurokinin-1 (NK-1) receptor has a strong affinity for substance P, which is expressed in vascular endothelial cells, muscles, neurons, and different types of immune cells [7]. NK-1 receptor antagonists may be centrally applied as antiemetics and analgesics and for treating urinary incontinence. These agents may also be used at the peripheral level for treating inflammatory bowel diseases, arthritis, inflammation, and cystitis [8–10].

Therefore, in view of the complexity and scope of receptor responses, a previous study used a rat model to assess the role of substance P and the activation of NK-1 receptors in the rostral ventromedial medulla, which is involved in hyperalgesia. This same study evaluated the role of substance P in thermal hyperalgesia and descending control of pain and found that this neuropeptide facilitated spinal hyperalgesia by interacting with gamma-aminobutyric acid type-A receptors and N-methyl D-aspartate (NMDA) [11].

Maropitant is an antagonist of neurokinin-1 (NK-1) receptors and blocks the pharmacological action of substance P on the central nervous system. This drug is indicated for patients who present with vomiting in several clinical situations. Some studies have shown that the NK-1 antagonist can reduce the minimum alveolar concentration (MAC) of sevoflurane in dogs [12–26].

The objective of this study was to evaluate the antinociceptive and cardiorespiratory effect of the continuous intraoperative infusion of maropitant with ketamine and lidocaine in female dogs undergoing unilateral radical mastectomy.

## 2. Materials and Methods

### 2.1. Animals

The study sample included 24 female dogs from the Veterinary Hospital of the State University of Santa Cruz (Universidade Estadual de Santa Cruz–UESC), Ilhéus, Bahia, Brazil. The study was approved by the Animal Research Ethics Committee under Protocol No. 006/2016.

The dog owners received information about the experimental procedure and authorized the inclusion of the animals in the study by signing an informed consent form.

The animals were clinically examined and subjected to a complete blood count, urea, creatinine, alanine aminotransferase (ALT), aspartate aminotransferase (AST), alkaline phosphatase, chest X-ray, and electrocardiogram.

The inclusion criteria were docile animals, normal laboratory test results, eligibility for the surgical/anesthetic procedure based on the electrocardiogram results, absence of pulmonary metastasis, classification of anesthetic and surgical risk as ASA I or II, and older animals with mild compensated cardiomyopathy.

Before the surgical and anesthetic procedure, the animals were food-fasted for 12 hours and water-fasted for 2 hours.

### 2.2. Experimental Design

In all the experimental groups, before administering the preanesthetic medication, a multiparameter monitor (T8 Bene View, Mindray Medical International Limited) was used to analyze the following parameters: body temperature (BT), respiratory rate (f), carbon dioxide concentration in the exhaled air (ETCO_2_), heart rate (HR), systolic blood pressure (SBP), mean blood pressure (MBP), and diastolic blood pressure (DBP). After the animals were subjected to the standard anesthetic protocol, preanesthetic morphine at 0.5 mg/kg IM was administered (Dimorf® 10 mg/mL, Cristália Produtos Químicos Farmacêuticos Ltda.), and the simple descriptive scale was used to evaluate the level of sedation. After 15 minutes of administering morphine as preanesthetic medication, trichotomy was performed in the abdomen and paws, and electrodes were placed on these body regions. A catheter was placed in the cephalic vein, and Ringer's solution with lactate (Lactated Ringer's Solution 500 mL, Fresenius Kabi Brazil Ltda.) was administered at a rate of 10 mL/kg/h as maintenance fluid therapy until the end of the surgical procedure. After fluid therapy, the animals were subjected to oxygen therapy 5 minutes before induction. Anesthetic induction was initiated with 5 mg/kg IV (Propotil® 1%, Bio Chimico®, Instituto Biochimico Indústria Farmacêutica Ltda.), and then, endotracheal intubation was performed. Anesthesia was maintained with inhalation isoflurane (Isofluoran®, inhalation solution, Instituto Biochimico Indústria Farmacêutica Ltda.). All animals received pressure-controlled mechanical ventilation. The anesthetic concentration of isoflurane was started with a standard value (1, 5 MAC), but according to the changes in clinical parameters such as the variation in blood pressure, we modify it with the decrease or increase.

### 2.3. Treatments

The dogs were randomly divided into two treatment groups (12 each group) by lottery. The animals received the following treatments in the intraoperative period and for 1 hour after the end of surgery using two syringe pumps (Injectomat Agilia infusion pump, Fresenius Kabi Brazil Ltda., Aquiraz, Ceará, Brazil).

Group lidocaine and ketamine (GLK): The LK group received, after anesthetic induction, ketamine bolus (1.0 mg/kg; Quetamina® injectable, Vetnil Indústria e Comércio de Produtos Veterinários Ltda.) and lidocaine bolus (1.5 mg/kg; 2% lidocaine hydrochloride, Hipolabor Farmacêutica Ltda.), and continuous infusion of ketamine (infusion rate, 10 mcg/kg/min) and lidocaine (infusion rate, 50 mcg/kg/min) was administered.

Group lidocaine, ketamine, and maropitant (GLKM): The LKM group received, after anesthetic induction, maropitant bolus (1.5 mg/kg; Cerenia®, maropitant citrate, injectable solution, Zoetis Indústria de Produtos Veterinários Ltda.), ketamine bolus (1.0 mg/kg), lidocaine bolus (1.5 mg/kg), and the continuous infusion of lidocaine (infusion rate, 50 mcg/kg/min), ketamine (infusion rate, 10 mcg/kg/min), and maropitant (infusion rate, 100 mcg/kg/h).

Unilateral radical mastectomy was performed in a standardized manner in all animals by an experienced surgeon. The antibiotic cephalothin sodium (30 mg/kg/IV; Ceflen®, cephalothin sodium, Agila Especialidades Farmacêuticas Ltda.) was administered during surgery.

### 2.4. Measures

The multiparametric monitor was used to measure BT (using an esophageal thermometer), f, SpO_2_, ETCO_2_, Etiso, SBP, MBP, DBP, and HR, at six intraoperative time points, as follows: M0, before any anesthetic procedure; M1, before the beginning of surgery, after induction; M2, after subcutaneous incision; M3, upon removal of the mammary chain; M4, during subcutaneous injection; M5, during skin suture; and M6, at the end of surgery.

The treatments were randomly divided by a team member who did not participate in anesthesia and postoperative evaluation. The levels of analgesia and sedation were measured by an evaluator who was unaware of the experimental groups in the following time points: T1, termination of continuous infusion; T2, 1 hour after termination of continuous infusion; T3, 2 hours after termination of continuous infusion; T4, 3 hours after termination of continuous infusion; T5, 4 hours after termination of continuous infusion; and T6, 5 hours after cessation of continuous infusion. These levels were determined using four scales: a simple descriptive scale, visual interactive and dynamic analog scale (DIVAS), numerical classification scale (NCS), and short-form Glasgow composite pain scale (GCPS-SF). The nociceptive mechanical threshold was determined using a digital analgesimeter (EFF-301, Insight Equipamentos).

### 2.5. Rescue Analgesia

In the postoperative period, the animals received rescue analgesia with 0.2 mg/kg/IM of morphine when the pain levels reached 33% of the maximum DIVAS score [27, 28] and/or >25% (6/24) of the maximum GCPS-SF score [28]. After the last evaluation, all animals were given 0.2 mg/kg/IM of meloxicam (Maxicam 2% injetável 50 ml, Ouro fino Saúde Animal Ltda., Brazil), 25 mg/kg IM of dipyrone (Santidor1 g/2 mL, Santista Laboratório Farmacêutica S.A., Brazil), and 4 mg/kg IM of tramadol (Cloridrato de tramadol 100 mg/2 mL, União Química Farmacêutica Nacional S/A, Brazil).

### 2.6. Statistical Analysis

The experimental design was completely randomized. Data were analysed using analysis of variance, subjected to a test of comparison of means using Student's *t*-test. The nonparametric Mann–Whitney and Friedman tests were used to analyze the variables that did not follow a normal distribution. The log-rank test was used to compare the survival curves (Kaplan–Meier curve) between the groups. All statistical analyses were performed at a level of significance of 5%.

## 3. Results

The time of surgery (GLK = 53.92 ± 15.71/GLKM = 64.92 ± 19.48) and the time of anesthesia (GLK = 70.75 ± 15.52/GLKM = 80.83 ± 20.24) showed no significant difference among the groups studied (*P* > 0.05). There were no differences in weight, propofol dose, surgical time, and extubation time between the groups. However, anesthesia complications required treatment in 33.3% of the studied animals. The most common complications were hypotension (25%) and bradycardia (8.3%), but there was no difference between groups (*P* > 0.05).

In animals with hypotension during surgery, dobutamine (5 mcg/kg/min) was administered until normotension was restored, and atropine (0.02 mg/kg) was administered in cases of bradycardia.

### 3.1. Parameters

The physiological parameters (BT, ETCO_2_, SpO_2_ Etiso, HR, SBP, MBP, and DBP) evaluated showed no statistical difference between the groups, and the means and standard deviations of the evaluated parameters are shown in Table 1.

### 3.2. Evaluation of Pain

**Figure 1:**
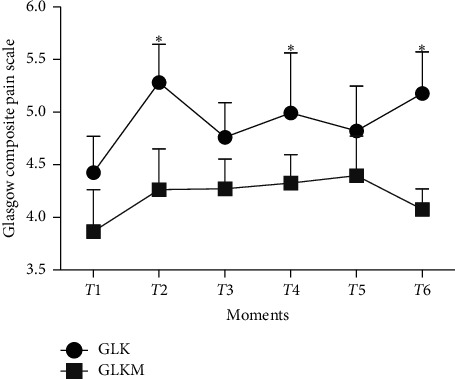
Mean ± standard error of the mean of the modified Glasgow composite scale for pain assessment in female dogs submitted to unilateral radical mastectomy, continuous infusion of lidocaine and ketamine (GLK), and continuous infusion of lidocaine, ketamine, and maropitant (GLKM) in the six moments of evaluation in the postoperative period (*P* < 0.01).

There were significant differences in pain between the groups after surgery using the MGCS-SF, and GLKM had lower pain scores (*P* < 0.01) (Figure 1).

**Figure 2:**
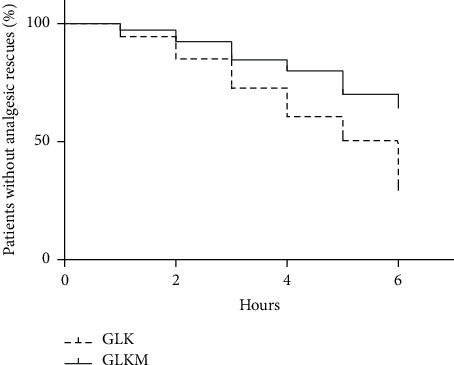
Survival rate in female dogs submitted to unilateral radical mastectomy, continuous infusion of lidocaine and ketamine (GLK), and continuous infusion of lidocaine, ketamine, and maropitant (GLKM) during the six hours of postoperative evaluation. For the survival test, the first rescue received by each animal was considered.

**Figure 3:**
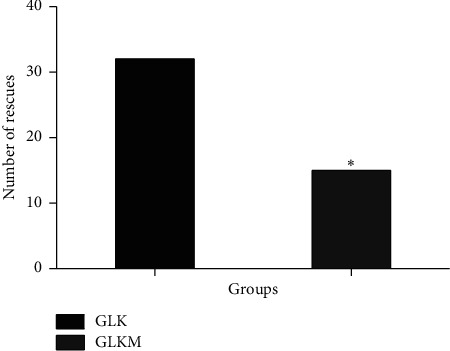
Total number of rescues in female dogs submitted to unilateral radical mastectomy, continuous infusion of lidocaine and ketamine (GLK), and continuous infusion of lidocaine, ketamine, and maropitant (GLKM) during the six hours postoperative evaluation, ^∗^*P* < 0.05.

There was a significant between-group difference in the survival curves for the number of rescue analgesia interventions required after surgery (*P* < 0.01) (Figure 2). The GLKM group had a better survival curve (*P* < 0.05) and a lower number of rescues (Figures 2 and 3).

**Figure 4:**
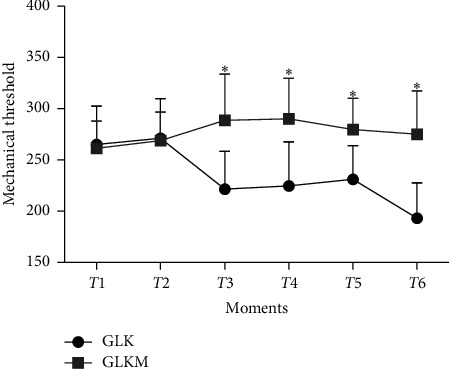
Mean ± standard error of the mean of using the digital analgesimeter at the secondary points for pain evaluation in female dogs submitted to unilateral radical mastectomy, continuous infusion of lidocaine and ketamine (GLK), and continuous infusion of lidocaine, ketamine, and maropitant (GLKM) during the six moments of postoperative evaluation.

Moreover, there were significant differences in the secondary mechanical nociceptive threshold between the two groups at T3, T4, T5, and T6 (*P* < 0.01) (Figure 4).

## 4. Discussion

In this study, unilateral radical mastectomy was performed in female dogs by removing the mammary chain and the subcutaneous and lymphatic tissue on one side of the midline. This extensive surgical resection causes much pain because of the degree of tension, flexibility of the involved muscles, and the amount of damaged tissue; however, the excision of the nodules improves the animal's quality of life, allows histological diagnosis, and reduces neoplastic progression [1, 2, 4].

In multimodal analgesia, the simultaneous administration of two or more analgesic drugs is safer and more effective than using one drug in the anesthetic protocol. The advantages of the bolus technique and continuous infusion of ketamine and lidocaine are well known, and this type of multimodal analgesia is widely used in mastectomies to achieve intraoperative and postoperative analgesia [29–31]. Therefore, this study helped assess improvements in analgesia using a new drug. Maropitant was chosen because this NK-1 receptor antagonist can block the pharmacological action of substance P in the central nervous system, which is involved in pain processing [12, 23, 24].

At present, maropitant is clinically indicated for treating acute vomiting in dogs; nonetheless, no studies to date demonstrated the antinociceptive potential of this drug in mastectomy. Therefore, this study is the first to use maropitant as an adjunct agent of analgesia in continuous infusion in unilateral radical mastectomy performed in female dogs.

It was found that inhalation of anesthetic isoflurane caused hypotension in the animals by reducing peripheral vascular resistance, myocardial depression, and blood pressure [32]. Therefore, dobutamine was used in some animals because of its positive inotropic effect, increasing the force of contraction and raising blood pressure. Dobutamine [33–35] stimulates beta-adrenergic receptors, increases cardiac output, and may decrease systemic vascular resistance [36]; this drug was used in hypotensive animals because of these effects.

Furthermore, atropine, which has an anticholinergic effect on muscarinic receptors, was used in the intraoperative period to reverse bradycardia [32].

Previous studies reported that the multimodal technique of continuous infusion of lidocaine produced analgesia and decreased heart rate [37, 38]. However, lidocaine induces the constriction or dilation of arterioles depending on the concentration, and the constriction effect predominates at low doses. Furthermore, lidocaine suppresses postoperative pain at peripheral sites [37, 38]. Lidocaine with ketamine reduces the MAC of inhaled anesthetics depending on the dose, maintaining cardiovascular stability. Furthermore, this drug combination provides effective analgesia by acting at different points of the nociceptive pathway [30].

Acute postoperative pain reaches maximum levels at 16 hours and becomes lower at 24 hours [39, 40]. The period of analgesia of ketamine + lidocaine and maropitant was 6 and 8 hours, respectively. Therefore, an evaluation time of 6 hours was used to evidence acute changes induced by the action of the drugs in the intraoperative period.

In the present study, there was a significant reduction in the postoperative pain intensity because the group receiving maropitant required a lower number of rescues. In the study using maropitant in ovariohysterectomy in cat, the results suggest that cats experienced greater comfort during the postoperative period because they required a lower number of analgesic rescues [10]. It was observed that the use of different methods of assessment of postoperative pain allowed veracity in the analysed values. In the present study, several physiological parameters and objective and subjective scales were used to measure postoperative pain in female dogs undergoing unilateral radical mastectomy [40, 41].

The GCPS-SF, which was used successfully to assess acute pain after the surgical procedure, has specific physiological and behavioral parameters and is essential for making comprehensive evaluations. Moreover, this scale produces consistent data and facilitates the analysis at different time points [28, 41].

There were significant differences in the survival curves and the number of rescue analgesia between the groups, demonstrating the efficacy of adjuvant maropitant in multimodal analgesia, as demonstrated in a study using maropitant in female dogs during noxious visceral stimulation of the ovary and ovarian ligament [42]. The hypothesis that postoperative nausea is an important clinical sign of pain was raised [13]. Maropitant is a potent antiemetic and, consequently, would influence the reduction in postoperative pain.

There was a difference in peripheral sensitization between the groups, and maropitant increased the mechanical nociceptive threshold. Peripheral sensitization is a combination of neurochemical changes arising from tissue injuries and inflammation in surrounding tissues, leading to secondary hyperalgesia. In it occurs the release of neuropeptides (substance P and calcitonin gene-related peptide), which increase the inflammatory response by local vasodilation, mast cell degranulation, and plasma extravasation [39, 40, 43]. It was found that hyperalgesia is attenuated in cases in which the NK-1 receptor is inhibited because the activation of NK-1 receptors produces hyperalgesia [44, 45]. Therefore, our results indicate that maropitant significantly inhibits peripheral sensitization.

## 5. Conclusions

This drug combination during surgery and for 1 hour after surgery increased patient comfort in the postoperative period, resulting in improved pain management and decreased use of opioids.

## Figures and Tables

**Table 1:** Variables (means ± SD) of the physiological parameters recorded during the study.

Variables	Groups	Intraoperative time points						
		M0	M1	M2	M3	M4	M5	M6
BT (°C)	GLK	38.7 ± 0.8	36.6 ± 0.8	36 ± 0.7	35.7 ± 0.7	35 ± 1.4	35 ± 0.8	34.9 ± 0.8
	GLKM	38.4 ± 1.2	36.8 ± 0.7	36.1 ± 0.9	36 ± 0.8	35.6 ± 1	35 ± 1.1	34.9 ± 1.2

SpO_2_ (%)	GLK	84.4 ± 12.3	97.6 ± 2.6	98.5 ± 1	98 ± 3.2	98.7 ± 1.5	98.7 ± 1.9	98.8 ± 2
	GLKM	88.2 ± 8.9	97.9 ± 2.2	98.7 ± 1.3	98.4 ± 1.6	98.4 ± 1.6	98.5 ± 1.2	98.8 ± 1.2

EtCO_2_ (mmHg)	GLK	32.6 ± 6.4	35.4 ± 6.5	33.8 ± 5.3	34.9 ± 8.4	35.6 ± 6.9	35.8 ± 7	36 ± 6.6
	GLKM	26.4 ± 11.2	35.2 ± 8.6	31.5 ± 6.8	33.7 ± 8.5	35 ± 7.7	32.6 ± 7.5	33.8 ± 8.3

Etiso (V%)	GLK	0	1.3 ± 0.2	1.4 ± 0.2	1.4 ± 0.2	1.3 ± 0.1	1.3 ± 0.2	1.3 ± 0.1
	GLKM	0	1.2 ± 0.2	1.4 ± 0.3	1.4 ± 0.2	1.3 ± 0.1	1.3 ± 0.2	1.2 ± 0.2

HR (bpm/min)	GLK	121.6 ± 20.7	102.1 ± 23.5	111 ± 24	109 ± 28.1	106.3 ± 25.2	105.6 ± 25.8	107.9 ± 21.4
	GLKM	123.2 ± 25.2	112.3 ± 24.6	106.7 ± 24.3	107.8 ± 24	114.3 ± 26.1	105.7 ± 23.9	107.6 ± 22.2

SBP (mmHg)	GLK	122 ± 38	88.9 ± 28	105.3 ± 36.6	106.7 ± 34	107.6 ± 30.6	113.8 ± 39.1	112.4 ± 34
	GLKM	124.1 ± 55.6	83.3 ± 24	98.3 ± 26.2	93.7 ± 37.2	97.9 ± 23.9	90.5 ± 27.9	97.1 ± 10.6

MBP (mmHg)	GLK	94.4 ± 30.7	62.8 ± 21.2	66.5 ± 18.8	77 ± 19.1	81.6 ± 19.9	88.8 ± 27.8	85.6 ± 30.5
	GLKM	91 ± 44.4	63.7 ± 16.2	76.8 ± 27.3	71.8 ± 13.7	69.6 ± 8.8	71.4 ± 19.7	71.3 ± 7.3

DBP (mmHg)	GLK	89 ± 81.4	52.8 ± 21.9	54.2 ± 18.6	64 ± 18.5	65.4 ± 19.8	75.4 ± 32	79.6 ± 28.3
	GLKM	88.2 ± 47	51.3 ± 17.1	66.4 ± 26.8	61.6 ± 12	57.6 ± 8.7	65.8 ± 28.4	64 ± 14.2

GLK: lidocaine and ketamine group; GLKM: lidocaine, ketamine, and maropitant group; BT: body temperature; SpO_2_: oxyhemoglobin saturation; EtCO_2_: concentration of carbon dioxide in exhaled air; Etiso: isoflurane concentration at the end of expiration; HR: heart rate; SBP: systolic blood pressure; MBP: mean blood pressure; DBP: diastolic blood pressure. M0: before any anesthetic procedure; M1: before the start of the surgical procedure; M2: after subcutaneous incision. M3: breast chain removal; M4: approach of the subcutaneous; M5: skin suture; M6: end of surgery. *P* > 0.05.

## Data Availability

The result data used to support the findings of this study are available from the corresponding author upon request.

## References

[1] Aguirre C. S., Minto B. W., Faria E. G., Horr M., Filgueira F. G. F., Nardi A. B. (2014). Anestesia convencional e técnica de tumescência em cadelas submetidas à mastectomia. Avaliação da dor pós-operatória. *Arquivo Brasileiro de Medicina Veterinária e Zootecnia*.

[2] Cassali G. D., Lavalle G. E., Ferreira E. (2014). Consensus for the diagnosis, prognosis and treatment of canine mammary tumors - 2013. *BJVP*.

[3] Horta R. S., Figueiredo M. S., Lavalle G. E., Costa M. P., Cunha R. M. C., Araújo R. B. (2015). Surgical stress and postoperative complications related to regional and radical mastectomy in dogs. *Acta Veterinaria Scandinavica*.

[4] Credie L. d. F. G. A., Luna S. P. L. (2013). Perioperative evaluation of tumescent anaesthesia technique in bitches submitted to unilateral mastectomy. *BMC Veterinary Research*.

[5] Kaka U., Cheng C. H., Meng G. Y. (2014). Electroencephalographic changes associated with antinociceptive actions of lidocaine, ketamine, meloxicam, and morphine administration in minimally anaesthetized dogs. *BioMed Research International*.

[6] Kaka U., Saifullah B., Abubakar A. A. (2016). Serum concentration of ketamine and antinociceptive effects of ketamine and ketamine-lidocaine infusions in conscious dogs. *BMC Veterinary Research*.

[7] Muñoz M., Muñoz M. F., Ayala A. (2017). Immunolocalization of substance P and NK-1 receptor in adipose stem cells. *Journal of Cellular Biochemistry*.

[8] Vink R., Gabrielian L., Thornton E. (2017). Review: the role of substance P in secondary pathophysiology after traumatic brain injury. *Frontiers of Neurology*.

[9] Quartara L., Maggi C. A. (1998). The tachykinin NK1 receptor. Part II: distribution and pathophysiological roles. *Neuropeptides*.

[10] Corrêa J. M. X., Soares P. C. L. R., Niella R. V. (2019). Evaluation of the antinociceptive effect of maropitant, a neurokinin-1 receptor antagonist, in cats undergoing ovariohysterectomy. *Veterinary Medicine International*.

[11] Lagraize S. C., Guo W., Yang K., Wei F., Ren K., Dubner R. (2010). Spinal cord mechanisms mediating behavioral hyperalgesia induced by neurokinin-1 tachykinin receptor activation in the rostral ventromedial medulla. *Neuroscience*.

[12] Borrego J. F., Huelsmeyer M. K., Pinkerton M. E. (2014). Neurokinin-1 receptor expression and antagonism by the NK-1R antagonist maropitant in canine melanoma cell lines and primary tumour tissues. *Veterinary and Comparative Oncology*.

[13] Marquez M., Boscan P., Weir H., Vogel P., Twedt D. C. (2015). Comparison of NK-1 receptor antagonist (maropitant) to morphine as a pre-anaesthetic agent for canine ovariohysterectomy. *PLoS One*.

[14] Ramsey D. S., Kincaid K., Watkins J. A. (2008). Safety and efficacy of injectable and oral maropitant, a selective neurokinin 1 receptor antagonist, in a randomized clinical trial for treatment of vomiting in dogs. *Journal of Veterinary Pharmacology and Therapeutics*.

[15] Mikawa S., Yamamoto S., Islam M. S. (2015). Anti-emetic drug maropitant induces intestinal motility disorder but not anti-inflammatory action in mice. *Journal of Veterinary Medical Science*.

[16] Fukui S., Ooyama N., Tamura J. (2017). Interaction between maropitant and carprofen on sparing of the minimum alveolar concentration for blunting adrenergic response (MAC-BAR) of sevoflurane in dogs. *Journal of Veterinary Medical Science*.

[17] Lorenzutti A. M., Martín-Flores M., Litterio N. J., Himelfarb M. A., Invaldi S. H., Zarazaga M. P. (2017). A comparison between maropitant and metoclopramide for the prevention of morphine-induced nausea and vomiting in dogs. *The Canadian Veterinary Journal = La Revue Veterinaire Canadienne*.

[18] Benchaoui H. A., Siedek E. M., De La Puente-Redondo V. A., Tilt N., Rowan T. G., Clemence R. G. (2007a). Efficiacy of maropitant for preventing vomiting associated with motion sickness in dogs. *Veterinary Record*.

[19] Benchaoui H. A., Cox S. R., Schneider R. P., Boucher J. F., Clemence R. G. (2007b). The pharmacokinetics of maropitant, a novel neurokinin type-1 receptor antagonist, in dogs. *Journal of Veterinary Pharmacology and Therapeutics*.

[20] Yalcin E., Keser G. O. (2017). Comparative eficacy of metoclopramide, ondansetron and maropitant in preventing parvoviral enterits-induced emesis in dogs. *Journal of Veterinary Pharmacology and Therapeutics*.

[21] Conder G. A., Sedlacek H. S., Boucher J. F., Clemence R. G. (2008). Efficacy and safety of maropitant, a selective neurokinin 1 receptor antagonist, in two randomized clinical trials for prevention of vomiting due to motion sickness in dogs. *Journal of Veterinary Pharmacology and Therapeutics*.

[22] Sedlacek H. S., Ramsey D. S., Boucher J. F., Eagleson J. S., Conder G. A., Clemence R. G. (2008). Comparative efficacy of maropitant and selected drugs in preventing emesis induced by centrally or peripherally acting emetogens in dogs. *Journal of Veterinary Pharmacology and Therapeutics*.

[23] de la Puente-Redondo V., Tingley F. D., Schneider R. P., Hickman M. A. (2007). The neurokinin-1 antagonist activity of maropitant, an antiemetic drug for dogs, in a gerbil model. *Journal of Veterinary Pharmacology and Therapeutics*.

[24] de la Puente-Redondo V. A., Siedek E. M., Benchaoui H. A., Tilt N., Rowan T. G., Clemence R. G. (2007). The anti-emetic efficacy of maropitant (Cerenia) in the treatment of ongoing emesis caused by a wide range of underlying clinical a etiologies in canine patients in Europe. *Journal of Small Animal Practice*.

[25] Alvillar B. M, Boscan P., Mama K. R. (2012). Effect of epidural and intravenous use of the neurokinin-1 (NK-1) receptor antagonist maropitant on the sevoflurane minimum alveolar concentration (MAC) in dogs. *Veterinary Anaesthesia and Analgesia*.

[26] Niyom S., Boscan P., Twedt D. C., Monnet E., Eickhoff J. C. (2013). Effect of maropitant, a neurokinin‐1 receptor antagonist, on the minimum alveolar concentration of sevoflurane during stimulation of the ovarian ligament in cats. *Veterinary Anaesthesia and Analgesia*.

[27] Lascelles B. D., Cripps P. J., Jones A., Waterman-Pearson A. E. (1998). Efficacy and kinetics of carprofen, administered preoperatively or postoperatively, for the prevention of pain in dogs undergoing ovariohysterectomy. *Veterinary Surgery*.

[28] Reid J., Nolan A. M., Hughes L., Lascelles B. D. X., Pawson P., Scott E. M. (2007). Development of the short-form Glasgow composite measure pain scale (CMPSSF) and derivation of an analgesic intervention score. *Animal Welfare*.

[29] Assumpção A. E., Naspolini B., Santalucia S., Heymanns A. C., Piovezan A. P. (2017). Avaliação de dois protocolos de analgesia transoperatória em cadelas submetidas à mastectomia unilateral total. *Acta Scientiae Veterinariae*.

[30] Bressan T. F., Monteiro E. R., Maciel N. S. (2013). Infusão contínua de lidocaína e/ou de cetamina como adjuntes durante anestesia inalatória em cães: revisão de literatura/Constant rate infusion of lidocaine and/or ketamine as adjuvants during inhalation anesthesia in dogs: literature review. *Revista Científica de Medicina Veterinária*.

[31] Muir W. W, Wiese A. J., March P. A. (2003). Effects of morphine, lidocaine, ketamine, and morphine-lidocaine-ketamine drug combination on minimum alveolar concentration in dogs anesthetized with isoflurane. *American Journal of Veterinary Research*.

[32] Flôres F. N., Moraes A. N. d., Oleskovicz N. (2008). Sulfato de atropina nos parâmetros hemodinâmicos e hemogasométricos de cães anestesiados com clorpromazina, dexmedetomidina e isoflurano. *Ciência Rural*.

[33] Valverde A., Giguére S., Sanchez L. C., Shih A., Ryan C. (2006). Effects of dobutamine, norepinephrine, and vasopressin on cardiovascular function in anesthetized neonatal foals with induced hypotension. *American Journal of Veterinary Research*.

[34] Park S. (2013). Prediction of hypotension in spinal anesthesia. *Korean Journal of Anesthesiology*.

[35] Pascoe P. J., Ilkiw J. E., Pypendop B. H. (2006). Effects of increasing infusion rates of dopamine, dobutamine, epinephrine, and phenylephrine in healthy anesthetized cats. *American Journal of Veterinary Research*.

[36] Rosati M., Dyson D. H., Sinclair M. D., Sears W. C. (2007). Response of hypotensive dogs to dopamine hydrochloride and dobutamine hydrochloride during deep isoflurane anesthesia. *American Journal of Veterinary Research*.

[37] Valverde A., Doherty T. J., Hernández J., Davies W. (2004). Effect of lidocaine on the minimum alveolar concentration of isoflurane in dogs. *Veterinary Anaesthesia and Analgesia*.

[38] Kawamata M., Sugino S., Narimatsu E. (2006). Effects of systemic administration of lidocaine and QX-314 on hyperexcitability of spinal dorsal horn neurons after incision in the rat. *Pain*.

[39] Basbaum A. I., Bautista D. M., Scherrer G., Julius D. (2009). Cellular and molecular mechanisms of pain. *Cell*.

[40] Gaynor S. J., Muir W. W. (2009). *Manual de Controle da Dor em Medicina Veterinária*.

[41] Matičić D., Stejskal M., Pećin M. (2010). Correlation of pain assessment parameters in dogs with cranial cruciate surgery. *Vetrinary Arhives*.

[42] Boscan P., Monnet E., Mama K., Twedt D. C., Congdon J., Steffey E. P. (2011). Effect of maropitant, a neurokinin 1 receptor antagonist, on anesthetic requirements during noxious visceral stimulation of the ovary in dogs. *American Journal of Veterinary Research*.

[43] Lamont L. A., Tranquilli W. J., Grimm K. A. (2000). Physiology of pain. *Veterinary Clinics of North America: Small Animal Practice*.

[44] Khasabov S. G., Simone D. A. (2013). Loss of neurons in rostral ventromedial medulla that express neurokinin-1 receptors decreases the development of hyperalgesia. *Neuroscience*.

[45] Hamity M. V., White S. R., Hammond D. L. (2010). Effects of neurokinin-1 receptor agonism and antagonism in the rostral ventromedial medulla of rats with acute or persistent inflammatory nociception. *Neuroscience*.

